# Characterizing the spatial distribution of multiple malaria diagnostic endpoints in a low-transmission setting in Lao PDR

**DOI:** 10.3389/fmed.2022.929366

**Published:** 2022-08-18

**Authors:** Isabel Byrne, Estee Cramer, Luca Nelli, Francois Rerolle, Lindsey Wu, Catriona Patterson, Jason Rosado, Elin Dumont, Kevin K. A. Tetteh, Emily Dantzer, Bouasy Hongvanthong, Kimberley M. Fornace, Gillian Stresman, Andrew Lover, Adam Bennett, Chris Drakeley

**Affiliations:** ^1^Department of Infection Biology, London School of Hygiene and Tropical Medicine, London, United Kingdom; ^2^Department of Biostatistics and Epidemiology, School of Public Health and Health Sciences, University of Massachusetts-Amherst, Amherst, MA, United States; ^3^School of Biodiversity, One Health and Veterinary Medicine, University of Glasgow, Glasgow, United Kingdom; ^4^Malaria Elimination Initiative, The Global Health Group, University of California, San Francisco, San Francisco, CA, United States; ^5^Department of Epidemiology and Biostatistics, University of California, San Francisco, San Francisco, CA, United States; ^6^Unit of Malaria: Parasites and Hosts, Institut Pasteur, Paris, France; ^7^Infectious Diseases Epidemiology and Analytics G5 Unit, Institut Pasteur, Paris, France; ^8^Center for Malariology, Parasitology and Entomology, Ministry of Health, Vientiane, Laos

**Keywords:** malaria, serology, active surveillance, passive surveillance, geostatistics, elimination

## Abstract

The epidemiology of malaria changes as prevalence falls in low-transmission settings, with remaining infections becoming more difficult to detect and diagnose. At this stage active surveillance is critical to detect residual hotspots of transmission. However, diagnostic tools used in active surveillance generally only detect concurrent infections, and surveys may benefit from sensitive tools such as serological assays. Serology can be used to interrogate and characterize individuals' previous exposure to malaria over longer durations, providing information essential to the detection of remaining foci of infection. We ran blood samples collected from a 2016 population-based survey in the low-transmission setting of northern Lao PDR on a multiplexed bead assay to characterize historic and recent exposures to *Plasmodium falciparum* and *vivax*. Using geostatistical methods and remote-sensing data we assessed the environmental and spatial associations with exposure, and created predictive maps of exposure within the study sites. We additionally linked the active surveillance PCR and serology data with passively collected surveillance data from health facility records. We aimed to highlight the added information which can be gained from serology as a tool in active surveillance surveys in low-transmission settings, and to identify priority areas for national surveillance programmes where malaria risk is higher. We also discuss the issues faced when linking malaria data from multiple sources using multiple diagnostic endpoints.

## Introduction

Through an intensification of their programmatic activities and an increased coverage of interventions (1), the Lao People's Democratic Republic (PDR) has seen substantial declines in malaria cases, with a fall in case incidence by 80% from 2016 to 2020, and no reported malaria deaths since 2018 (2). Transmission is very low in northern Lao PDR, with *P. vivax* cases making up the majority of malaria burden (1, 3). The nation aims to eliminate *Plasmodium vivax* and *Plasmodium falciparum* from northern areas by 2025, and all species nationwide by 2030 (2, 4).

As countries near elimination and transmission declines, they experience characteristic shifts in malaria epidemiology. Substantial areas become malaria-free, and malaria risk becomes increasingly heterogenous and geographically or demographically clustered (5, 6), with cases becoming more difficult to detect and diagnose. Here, passive surveillance systems become inadequate as the sole method of data collection to inform population-level burden estimates (5–7). Estimates may be biased by different treatment-seeking behavior in high-risk populations, and the quality of record keeping may vary between health facilities or administrative regions (8). Passive surveillance also fails to detect asymptomatic individuals, which act as parasite reservoirs and contribute to continued transmission, even in low-transmission settings (9–11). At this stage it is important to find remaining clusters of transmission where infection remains high in order to target resources effectively (6, 7). Finding these residual foci of transmission involves actively seeking out infections, often through screening or surveying populations irrespective of malaria symptoms. This active surveillance can complement passive surveillance, and can play a role in interrupting transmission as countries near elimination (11). Active surveillance surveys for malaria are typically cross-sectional and involve sampling communities using RDT diagnostics, often collecting valuable added information on cases and specific populations who are at higher risk of infection (1, 5). As prevalence drops in elimination settings, passive and active surveillance surveys face the challenge of detecting sufficient concurrent infections to obtain a full picture of the epidemiology within a population, even when robust sampling-strategies are applied (1). In these situations, more sensitive diagnostic tools are needed to improve burden estimates and understand whether transmission is ongoing (12).

Serological assays are useful in such low-transmission settings. Rather than solely capturing concurrent infections, serology measures specific antibody responses which reflect previous exposure to pathogens. Different malaria antigens elicit different antibody responses, each of which last for different durations in the immune system (13, 14). Longitudinal research into antibody kinetics has resulted in a highly informative and diverse set of biomarkers being identified for *P. vivax* and *P. falciparum* infections. These characterize an individual's exposure history, and when sampled en masse, can provide information on the short-, medium- and long-term trends in malaria transmission in a population, highlighting changes in transmission over longer durations than PCR-based surveys (13, 15–18). Serological methods have been shown to be a useful complementary tool where traditional parasitological tools are not sensitive enough to estimate recent and active exposure and transmission intensity in low-transmission settings (11, 19). Serological multiplex bead assays (MBA) make serological surveys operationally feasible and can be added as a supplemental aspect of population surveys, as they can measure a broad range of immune responses from a single blood spot (14, 20). Measuring population-level serological responses using MBA can be useful in showing spatial heterogeneity of malaria exposure, finding clustering or hotspots of transmission and to predict receptive areas at risk of outbreaks (12, 21, 22).

Geostatistical methods are increasingly being applied in disease research to relate infection metrics with environmental, spatial and temporal covariates (14). In malaria research there are numerous recent analyses projects involving geostatistical mapping of malaria incidence, prevalence and other metrics (23, 24). In low-resource and/or low-transmission settings where infection data is sparse and transmission becomes more spatially heterogeneous, geostatistical mapping can identify and highlight areas where risk is more concentrated and may require targeted interventions from programme implementers (23, 25). Alongside the useful predictions of disease burden, geostatistical analyses can also identify areas of uncertainty in predictions, which can be used to prioritize future data collection (23).

Integrating geostatistical methods with serology data collected during active surveillance surveys provides an opportunity to characterize the spatial distribution of recent and historic exposure to different malaria antigens in a low-transmission setting. We ran blood samples from a 2016 active surveillance population survey (1) in northern Lao PDR using a serology MBA to gain an understanding of current and historic exposures to *P. vivax* and *P. falciparum*. We additionally used passive surveillance (case incidence) data collected from health facilities in the same districts (3) to compare serology and PCR-derived prevalences with burden estimates from active surveillance at the health facility catchment- level. We fit geostatistical models to predict historic and recent exposures to *P. vivax* and *P. falciparum*. We aimed to highlight the additional information which can be leveraged from serology as a complementary tool to passive and active surveillance in low-transmission settings, and to identify areas with elevated risks of malaria transmission requiring prioritization for national surveillance programmes.

## Methods

### Study site

**Figure 1:**
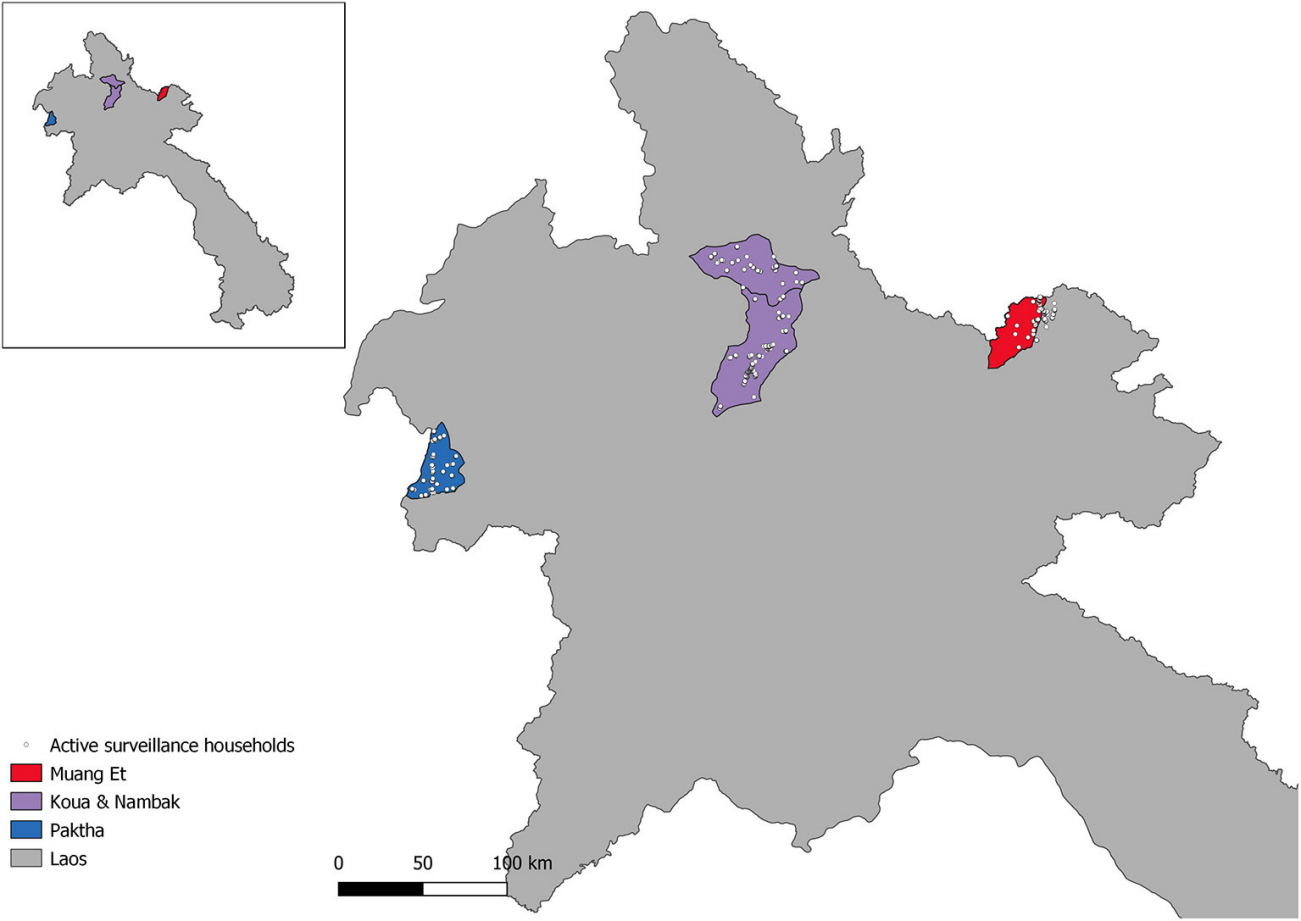
Map of Northern Lao PDR with active surveillance study households and districts of the study.

The active surveillance survey was conducted in four districts (Et, Paktha, Nambak and Koua) of northern Lao PDR (Figure 1), which are situated in four northern provinces (Bokeo, Huaphanh, Phongsaly and Luang-Prabang). The survey was conducted following the rainy season, between September and October 2016. The districts were chosen to focus on areas of malaria hotspots, and to ensure representation by surveying from diverse epidemiological settings (1). At the time of the study *P. vivax* was endemic and *P. falciparum* had reached historical lows in these provinces (3). It is a mountainous region characterized by a diverse climate, with low population density and limited access to roads (1, 3). The region shares borders with China, Myanmar, Thailand and Vietnam.

### Study design

The passive surveillance data were gathered according to Rerolle et al. (3). Briefly, a retrospective review of malaria registries between 2013 and 2016 was performed at health-facilities in the active survey districts. The data gathered from the registries included testing for malaria by RDT or microscopy, date, species-specific test results, village location and demographic variables including age and gender of those tested. For the present study, the dataset was sub-set to include records from 2016.

The survey data were collected according to Lover et al. (1). Briefly, a stratified two-stage cluster-sampling design was used. In each district catchment 25 survey clusters of 50 individuals were chosen for sampling, providing 1,250 participants per district and 5,000 overall. The district malaria office catchments were determined from local-level health office lists and did not always conform to official administrative boundaries. As a result, in the eastern district of Et a portion of the households fall outside of the official administrative boundaries (Figure 1). All residents and visitors who were over 18 months old and had spent the previous night in the household were invited to participate. Written or thumbprint consent was obtained from all participants. Upon informed consent, eligible individuals were tested with CareStart Ag Pf/Pv (SD Bioline, Cat #05FK80) rapid diagnostic tests and treated according to national guidelines if found positive. Four blood spots were collected on Whatman 903 “Protein Saver” sample cards (GE Healthcare; Cardiff UK). These were dried and cooled in refrigerators until subsequent analysis. Geographic coordinates were collected for all participating households.

### Laboratory procedures

The chemical coupling of 17 *P.falciparum* and *P.vivax* antigens (Supplementary Table 1) to MagPlex© beads (Luminex Corporation, TX, USA) were previously optimized *via* titration as described by Wu et al. (26). 3 mm punches of one blood spot from each Whatman 903 Protein Saver card were eluted 1:100 in buffer B [1xPBS, 0.05% Tween, 0.5% BSA, 0.02% sodium azide, 0.1% casein, 0.5% polyvinyl alcohol (PVA), 0.5% polyvinyl pyrrolidone (PVP)] containing 15.25 ug/mL *E.coli lysate* to prevent non-specific binding to antigens expressed in *E.coli*. 50 uL of each 1:100 blood spot elution was co-incubated with 1,000 coupled beads per antigen and specific IgG antibody binding was detected using 50 uL of R-PE conjugated secondary antibody [Jackson Immunoresearch 109-116-098: Goat anti-human Fcy-fragment specific IgG conjugated to R-Phycoerythrin (R-PE)] diluted 1:200 as described previously (26). Background-adjusted median fluorescent intensity (MFI) of wells achieving at least a 35-bead count per antigen were measured using a Luminex MAGPIX bioanalyzer and xPONENT software (version 4.2). *P. falciparum* positive control (NIBSC, 10/198), *P. vivax* positive control (72/96, NIBSC) and a curve of pooled hyperimmune *P.falciparum* sera (CP3, LSHTM) were included in singlicate on each test plate to assess interplate variability. 96 malaria-naïve sera from the UK (Public Health England 2016) were assayed at 1:100 using the same method.

PCR testing was performed on dried blood spots as described by Lover et al. (1), using previously described methods (27). Four blood spots were lysed on 96-well plates overnight at 4°C with 150 μl per well of HBS 1X/Saponin 0.5%. Samples were washed twice with HBS 1X and Instagene® Matric resin (Bio-Rad, Singapore) was used according to manufacturer's instructions to extract DNA. In order to limit the presence of inhibitors an additional centrifugation step (4,000 rpm, 20 min) was added, and a final volume of 50 μl of the supernatant was transferred into a new 96-well plate. Extracted DNA samples were screened for the presence of *Plasmodium* DNA using a qualitative real-time PCR assay which targeted *Plasmodium cytochrome b* gene (27). Positive samples were analyzed for *Plasmodium* species *P. falciparum, P. vivax, P. ovale* and *P. malariae*, using four real-time PCR assays (27).

### Statistical analysis

Individuals were classified as seropositive or seronegative for historical and recent exposures to *P. vivax* and *P. falciparum* based on their responses to the antigens in Table 1. The antigens in Table 1 were chosen based on their known longevity in the immune system. Pf/PvMSP119 and Pf/PvAMA1 are known to persist in the blood for many years and can be used as a proxy for any previous exposure in an individual's lifetime (14). Etramp5.Ag1 and PvEBPII are known to be shorter-lived and are used here to represent exposure within 6–9 months (17, 27).

**Table 1:** Table of malaria antigens used to define P. falciparum and P. vivax exposures, broken down by Plasmodium species. Including Plasmodb ID and reference source.

**Species**	**Antigen**	**Description**	**Exposure period**	**Plasmodb ID**	**Reference**
*P.falciparum*	PfAMA1	Apical membrane antigen 1	Historic	PF3D7_1133400	(44)
*P.falciparum*	PfMSP1_19	Merozoite surface protein 1-19	Historic	PF3D7_0930300	(45)
*P.falciparum*	Etramp 5 Ag 1	Early transcribed membrane protein 5 antigen (exon) 1	Recent	PF3D7_0532100	(46), Tetteh K unpublished
*P.vivax*	PvMSP119	Merozoite surface protein 1-19	Historic	PVX_099980	(47, 48)
*P.vivax*	PvAMA1	Apical membrane antigen 1	Historic	PVX_092275	(49)
*P.vivax*	PvEBPII	*P*. *vivax* erythrocyte binding protein	Recent	PVX_110810	(50–52)

We used unsupervised machine learning K-means clustering algorithms on each antigen separately to group samples into positives or negatives based on their MFI values. The optimal numbers of clusters for each antigen was determined using within-cluster sum of squares and average silhouette testing (28). Historic exposure to *P. vivax* was calculated as a combined exposure to PvMSP119 and PvAMA1. If an individual was classified as seropositive to one or both of these antigens, they were classified as being seropositive to historical exposure to *P. vivax*. Recent exposure to *P. vivax* was determined by seropositivity to PvEBPII. If an individual was positive for PvEBPII, they were classified as recently exposed to *P. vivax*. Historic exposures to *P. falciparum* were calculated as a combined exposure to PfMSP119 and PfAMA1. If an individual was classified as seropositive to one or both of these antigens, they were classified as being seropositive to historical exposure to *P. falciparum*. Recent exposure to *P. falciparum* was defined by seropositivity to Etramp5Ag1 antigens. If an individual was positive for Etramp5Ag1, they were classified as recently exposed to *P. falciparum*. Age-stratified seroprevalence was estimated for proportional age groups for each species and each exposure.

### Spatial analysis

The spatial distribution of exposure risks were assessed using geostatistical methods. Satellite-derived potential spatial and environmental covariates were assembled to assess associations with exposure risk. Covariates included topographic measures, distance to land cover types, forest cover and forest loss, population density, accessibility, and climactic variables (Supplementary Table 1). Pearson correlation coefficients were calculated, and highly correlated variables (correlation coefficient > 0.8) were excluded from the final dataset. All covariates were resampled to 250 m for predictions.

Geostatistical models of household seroprevalence for each species exposure were fit separately for the 1,402 households in the active surveillance. The models were fit within a Bayesian framework where *p*(*x*_*i*_) denotes seroprevalence at locations *x*_*i*_, *i* = 1, …, *n*, the number of positive households *Yi* out of *Ni* people sampled follows a binomial distribution:


$$\begin{array}{l} Yi|P\left(x_{i}\right)\sim \;Binomial\left(N_{i},P\left(x_{i}\right)\right),\\ logit\left(P\left(x_{i}\right)\right)=\beta_{0}+d{\left(x_{i}\right)}^{\prime }\beta +w_{i} \end{array}$$

Where β_0_ denotes the intercept, ***d(x***_***i***_***)*****′β** denotes a vector of location specific covariate effects (within active survey district boundaries) and *w*_*i*_ represents the spatial effect. The spatial effects were modeled as a Matérn covariance function using the stochastic partial differential equation (SPDE) approach in Integrated Nested Laplace Approximation (R-INLA) (29). The intercepts and fixed effect coefficients were fitted with weakly informative priors of Normal (0,100). Deviance information criteria (DIC) were used to assess the final models. A continuous surface of prevalence predictions for the active survey districts were extracted as the mean of posteriors of the predictions for each model. For the eastern district of Et, we extended the predictions to include the neighboring district of Xienghor, as a portion of the survey households fell close to or over the official district border. Exceedance probabilities for a 20% seroprevalence threshold for *P. vivax* and a 5% seroprevalence threshold for *P. falciparum* exposures were also extracted. These metrics represent the probability of the seroprevalence in each location exceeding its given threshold, where probabilities around 0.5 represent high uncertainty around the threshold (30). The upper and lower limits of the 95% credible intervals were also extracted to visualize uncertainty. Prevalence predictions, exceedance probabilities and upper and lower limits of the 95% credible intervals were converted to raster files and visualized in QGIS.

To evaluate health facility catchment-level seroprevalences and PCR prevalences, we estimated catchment areas for all health facilities from an official Lao PDR Ministry of Health list (31) of 190 health facilities in the country. A friction surface map of motorized travel time in Laos (32) was used to create 190 rasters of travel time to each health facility. The travel time rasters were then combined into a final raster of minimum travel time to each of the health facilities and converted into a polygon shapefile of catchment boundaries based on lowest-travel time to health facility. The catchment areas were estimated using script adapted from Weiss et al. (32) in RStudio version 1.4. Household seroprevalence estimates were linked to catchment boundaries using QGIS, and catchment-level seroprevalences and PCR prevalences were calculated for each species exposure. Positive RDT and microscopy levels per capita for 2016 were calculated from the estimated population size for each catchment.

### Ethics

Approvals for the field surveys (PI: Adam Bennett) were obtained from UCSF (approval 16-19649; 7-20-2016) and the Lao National Ethics Committee for Health Research, Lao Ministry of Health (approval 2016-014; 8-22-2016). Both approvals included provisions for future analysis of serological markers of malaria exposures.

## Results

The results from the retrospective survey of 2016 passive surveillance records from the study regions are presented in Table 2. In total 343 *P. vivax* cases and 36 *P. falciparum* cases were confirmed by RDT. 23 *P. vivax* cases and 12 *P. falciparum* cases were confirmed by microscopy.

**Table 2:** Numbers of positive cases by species confirmed by RDT and microscopy from passive surveillance (health center) 2016 records.

**District**	**Health Center**	**RDT Pf +ve**	**RDT Pv +ve**	**Microscopy Pf +ve**	**Microscopy Pv +ve**
Khua	Buamaphan	5	60	1	2
Khua	Lardsang	1	1	0	0
Khua	Nayang	3	37	0	2
Khua	Vikocmueng	0	0	0	0
Nambak	Khunolum	0	0	0	0
Nambak	Makpouk	18	100	0	0
Nambak	Muengteng	0	0	0	0
Nambak	Numnga	1	1	0	0
Nambak	Numthuan	0	2	0	0
Et	Naphieng	0	118	0	0
Et	Xiengkhoun	3	13	11	19
Paktha	Hardsa	0	1	0	0
Paktha	Houisat	0	0	0	0
Paktha	Jiengtong	0	3	0	0
Paktha	Kengphak	0	0	0	0
Paktha	Kiewlom	0	2	0	0
Paktha	Konteum	5	5	0	0

Table 3 provides descriptive statistics on the participants involved in the active survey. 5,084 individuals were samples from 1,402 households, with an average of 3.6 samples per household.

**Table 3:** Age range and gender breakdown of participants in active survey.

**Characteristic**	***n***	**% total (95% CI)**
Sex		
Male	2,380	46.8 (45.7–48.0%)
Female	2,702	53.2 (52.0–54.3%)
Age Group		
<5	273	5.4 (4.6–6.3%)
5–15	1,198	23.6 (21.9–25.3%)
> 15	3,611	71.0 (73.0–76.1%)

The mean MFI values for the antigens used to define historic and recent exposure to *P. falciparum* and *P. vivax* are as follows: PfMSP119 541.26 (±1334.88); PfAMA1 723.33 (±1785.43); Etramp5.Ag1 99.31 (±183.71); PvMSP119 169.272 (±181.789); PvAMA1 483.1 (±1772.88); PvEBPII 371.64 (±860.05). The range of individuals' MFI values by positivity and age are presented in Supplementary Figures 1A,B.

Table 4 presents the results of the PCR and serological exposures from the active survey including the number of individuals and households sampled per district and the number positive for PCR and serological exposure to *P. vivax* and *P. falciparum*.

**Table 4:** Numbers of individuals and households tested in active surveillance survey per district, with number positive for P. vivax and P. falciparum by PCR and serology (with prevalence in brackets).

**Districts**	**Individuals sampled**	**Households sampled**	**Serology *P. vivax* historic +ve**	**Serology *P. vivax* recent +ve**	**Serology *P. falciparum* historic +ve**	**Serology *P. falciparum* recent +ve**	**PCR *P. vivax* +ve**	**PCR *P. falciparum* +ve**	**RDT *P. falciparum +ve***	**RDT *P. vivax +ve***
Paktha	983	357	308 (0.31)	88 (0.09)	52 (0.05)	21 (0.02)	3 (0.003)	2 (0.002)	0 (0.000)	0 (0.000)
Khua and Nambak	2,393	683	470 (0.2)	46 (0.02)	46 (0.02)	24 (0.01)	17 (0.007)	3 (0.001)	0 (0.000)	0 (0.000)
Et	1,418	362	289 (0.2)	52 (0.04)	19 (0.01)	10 (0.00)	3 (0.002)	3 (0.002)	0 (0.000)	0 (0.000)

*+ve = positive*.

**Figure 2:**
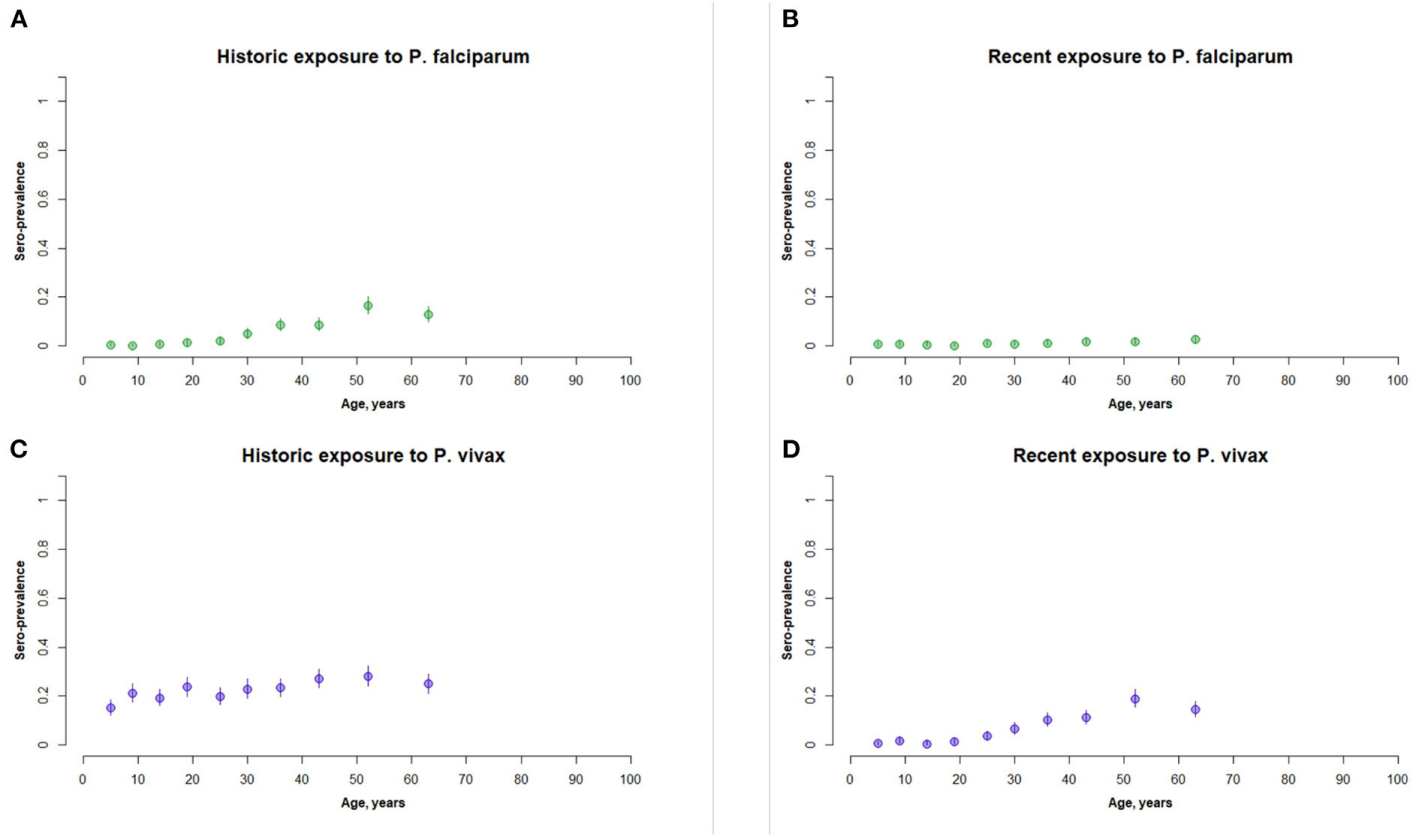
Panel of age stratified seroprevalence graphs for **(A)** historic exposure to P. falciparum, **(B)** recent exposure to P. falciparum, **(C)** historic exposure to P. vivax, and **(D)** recent exposure to P. vivax. This represents the full active survey population (*n* = 5,084).

At the survey-level seroprevalences for exposures to *P. vivax* were higher (0.22 for historic and 0.07 for recent) than *P. falciparum* (0.03 for historic and 0.01 for recent). The age-stratified seroprevalences for each species exposure are shown in Figure 2. Exposure was positively associated with increasing age for historic exposure to *P. falciparum* and recent exposure to *P. vivax*. These increases began at around 25 years of age for both exposures. Recent exposure to *P. falciparum* was very low across all age groups.

The PCR survey also found higher case numbers of *P. vivax* (*n* = 23) than *P. falciparum* (*n* = 8). Of the 23 positive *P. vivax* PCR cases in the active survey, seven were classified as positive for recent exposure to *P. vivax* (30%). Of the eight positive *P. falciparum* PCR cases in the active survey, one was classified as positive for recent exposure to *P. falciparum* (12.5%). The central districts of Khua and Nambak recorded the highest number of positive PCR *P. vivax* cases. *P. falciparum* PCR case numbers were similar across the three study areas.

**Figure 3:**
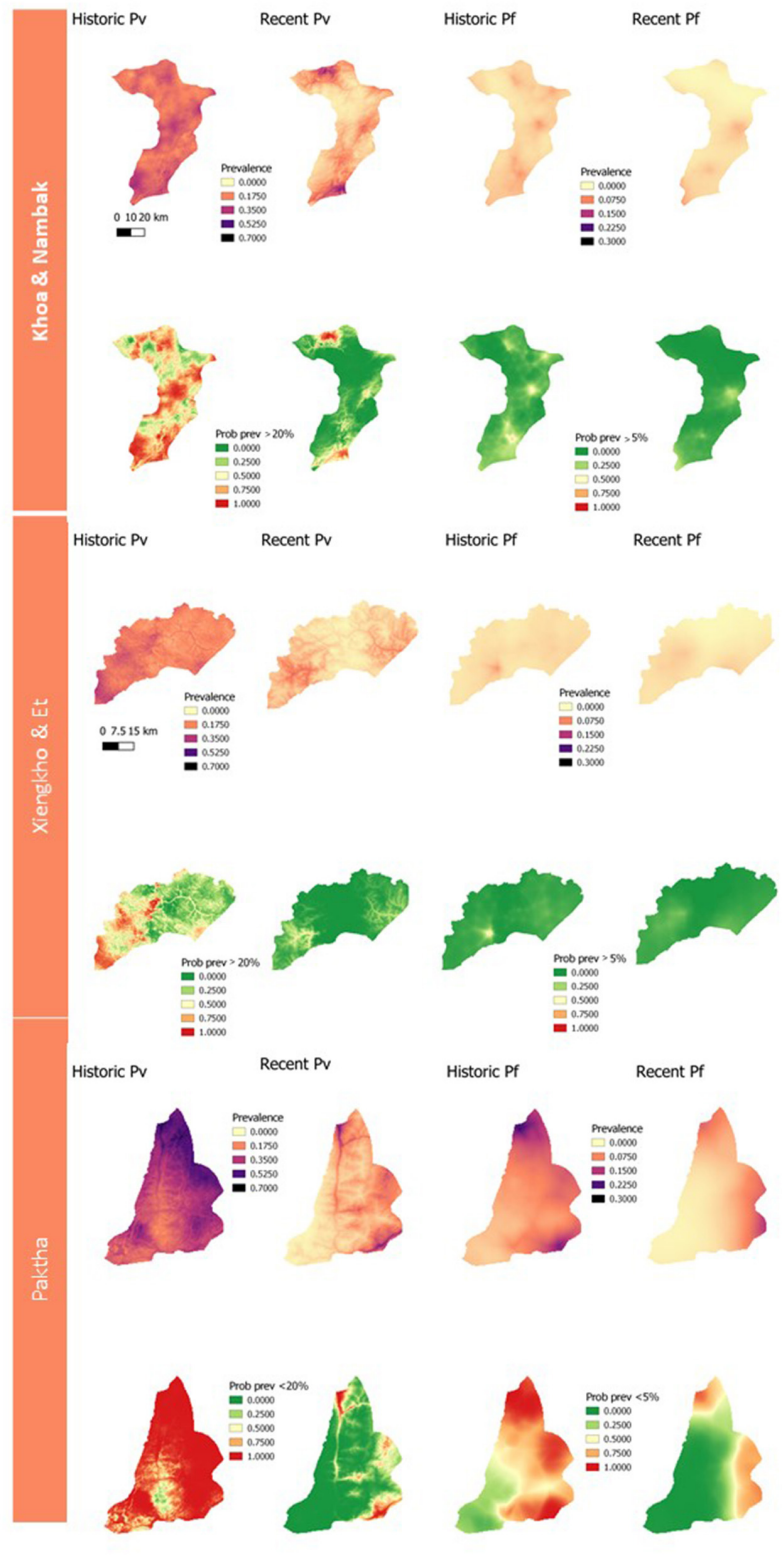
Panel of geostatistical maps of predicted seroprevalences and exceedance probabilities to historic and recent P. vivax and P. falciparum exposure for each district. Exceedance probabilities are on the bottom row of each district panel and refer to the probability of a location having >20% predicted prevalence for P. vivax exposures, and >5% for P. falciparum exposures.

Figure 3 presents the results of the geostatistical modeling for household seroprevalences to *P. vivax* and *P. falciparum* exposure. The environmental and spatial covariates included in the final geostatistical models are listed in Supplementary Table 3.

**Figure 4:**
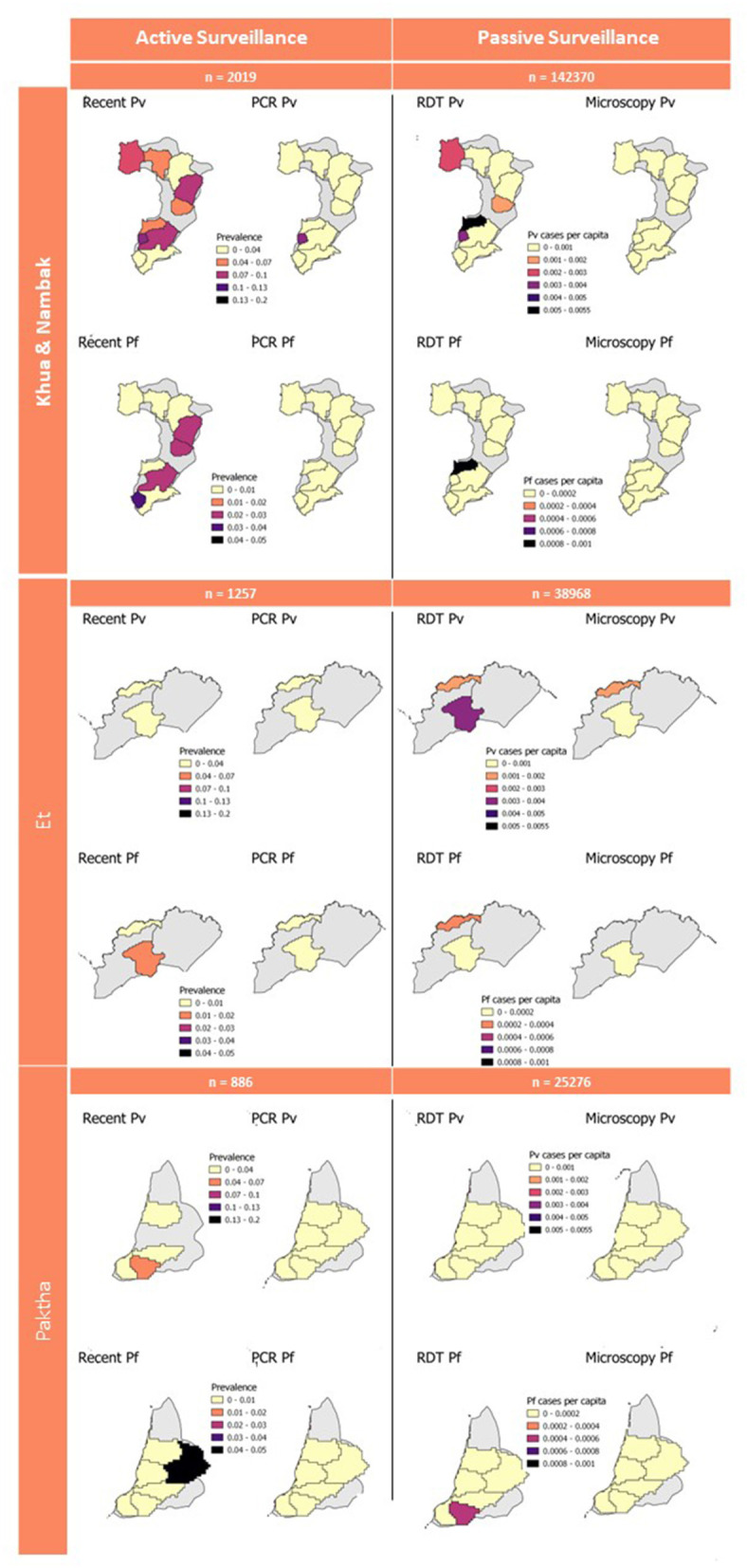
Active and passive survey diagnostic results aggregated to catchment level. Gray background represents full district area used for geostatistical predictions in Figure 3. Sample numbers for active surveillance are number of samples from active survey. Sample numbers for passive surveillance are the 2016 population within health facility catchments which was used to calculate cases per capita.

The estimation of catchment size resulted in 190 catchments across Lao PDR (Supplementary Figure 1). Due to the imperfect alignment of the passive surveillance data (3) and the active surveillance survey (1), 286 of the cross-sectional survey households (1,008 samples) did not fall within the catchment boundaries. This resulted in a significant reduction in the sample size for estimating PCR and seroprevalences at the catchment-level. RDT and microscopy cases per capita were calculated using the estimated population size for each health facility for 2016 (3). The catchment-level seroprevalences for recent exposure to *P. vivax* and *P. falciparum* and RDT and microscopy cases per capita are presented in Figure 4.

## Discussion

This study has demonstrated the use of integrating serology into active surveillance projects to provide additional information on historic and recent exposures to malaria. We have shown how geostatistical modeling with remote sensing-derived environmental variables can be used to predict and characterize the distribution of malaria exposures, and how these can be used to highlight priority areas for added data collection or targeted interventions. We found that historical exposures to *P. vivax* and *P. falciparum* were more widespread in northern Lao PDR, with recent exposures being more focally distributed, as is expected in an elimination setting. Additionally, we showed how retrospectively collected passive surveillance data can be linked to active surveillance data which were not collected in alignment.

The active surveillance survey involved rigorous population sampling which was powered to estimate PCR-based prevalences of malaria in northern Lao PDR as the country prepared for elimination (1). They found very low numbers of asymptomatic *Plasmodium* infections, with higher numbers of *P. vivax* (28 total, 0.005 prevalence) and lower numbers of *P. falciparum* (eight total, <0.000 prevalence). The seroprevalence rates estimated in this study for recent exposure to *P. vivax* and *P. falciparum* were higher than these PCR rates but followed similar trends. The higher numbers of *P. vivax* cases compared to *P. falciparum* detected by Lover et al. (1) and recorded by the health facilities (Figure 4) are consistent with our seroprevalence estimations. The almost complete absence of *P. falciparum* PCR-cases and very low numbers recorded in 2016 by health facilities are also aligned with our very low estimation of recent exposure to *P. falciparum*, suggesting that northern Lao PDR was close to eliminating this species during the year of study. We classified recent exposure to *P. vivax* and *P. falciparum* in 30 and 12.5% of PCR-confirmed cases. This shows that our classifications to recent exposure by serology detect some, but not all concurrent infections. Additionally, the findings show that antigens used for these classifications may be useful indicators of current as well as recent exposure. However, the very low sample size of PCR-confirmed cases for both species mean these findings cannot be confirmed in this study. Our findings provide evidence that at a broad level, our serological estimations reflect what is seen in both PCR-based and routine clinical case management. They also highlight the added information which can be extracted from active surveillance samples with the operationally feasible addition of MBA technology.

The age-stratified seroprevalence curves of recent exposure for *P. vivax* and historic exposure for *P. falciparum* are consistent with our expectations for a low-transmission setting in the Greater Mekong Region (GMR). Here, and in Lao PDR, malaria transmission is heterogenous, and transmission is more intense in forested areas (33, 34). Malaria exposure is largely an occupational hazard for forest workers, with transmission higher in remote forested areas, logging camps and plantations where conventional malaria vector control tools are inefficient (33). In these populations, exposure typically begins at around 20 years old when forest work begins (35–39). Historical exposures for *P. falciparum* increase with age, as is expected as transmission was higher during the lifetimes of older populations. We are therefore confident in our methodology using k-means algorithms for classifying seropositive and seronegative individuals, in the absence of international standards for estimating malaria seropositivity in a population. This characteristic age-stratified curve was not seen for historic exposure to *P. vivax*. One explanation for this is that it may be artifact of sampling bias, where the true highest-exposed groups were less likely to be captured at home and more likely to be working outside of the home (8, 34, 40).

The geostatistical mapping of serology data in this study allowed for the characterization of the spatial heterogeneity of remaining foci of *P. vivax* and *P. falciparum* infection. This mapping enables the estimation of seroprevalence at health-decision making units (probabilities of being over a given threshold), alongside measures of uncertainty (23). If taken up by National Malaria Control Programmes, this could allow prioritization of elimination efforts to the areas which they would be most impactful. In this study we arbitrarily chose thresholds of 20% exposure to *P. vivax* and 5% exposure to *P. falciparum* due to the local epidemiology of these species at the time of data collection. In future exercises where geostatistical maps of serology data are used to inform elimination programmes closer to the time of data collection, these thresholds could be set by the programmes according to their own criteria. The prediction maps and maps of exceedance probabilities follow the trend seen in the population-level classifications of seropositivity. We predicted higher and more widespread exposures to historical antigens and lower, more focalised exposures to recent antigens. In addition, predictions for recent and historic *P. vivax* burdens were higher than those for *P. falciparum*.

Despite the value of the geostatistical mapping, we faced a number of limitations. The survey households were clustered within three distinct areas in northern Lao PDR (Figure 1). Geostatistical mapping works by exploiting correlation between nearby data points and utilizing environmental and spatial covariates to produce estimates on a continuous surface (41, 42). Predicting at too far of a spatial range from sampling points results in higher uncertainty around predictions, which is less useful for public health programmes. Therefore, we limited the geostatistical mapping predictions to the districts the samples were collected from and did not produce larger province-level prediction maps. The spatial distribution of sampling points is an important consideration to take into account for projects planning to produce geostatistical maps, where a wider distribution of points can allow for predictions over larger spatial scales. The potential for relapses of *P. vivax* due to the reactivation of hypnozoite stage parasites should also be considered when interpreting the *P. vivax* maps and seroprevalence estimates. While our seroprevalence estimates and prediction maps followed the expected trend of declining in size and becoming more focal as exposure transitioned from historic to recent, the possibility of recurrent infections inflating exposure estimates and predictions should still be acknowledged. Differentiating between new and relapse cases remains challenge in *P. vivax* research and should be taken into account in mapping projects especially, as antibody production may occur in a different location to the original site of exposure (43).

While we have demonstrated a methodology for linking passively collected health-facility data to active surveillance data in the absence of health-facility catchment area boundaries, this aspect of the study had several important limitations. Firstly, the summary statistics presented for these areas are likely subject to modifiable areal unit problems. The list of health facilities used to create the catchment areas (31) (Supplementary Figure 1) was more expansive than that used by the retrospective review of malaria registries (3). This may be explained by the regular updating of the official online roster of health facilities, resulting in more locations listed in 2021 than during the year of the survey. As a result, some areas within the active surveillance districts were broken into catchments for health facilities which we did not have records for. 286 households comprising 1,008 samples were located in these areas and were thus lost from the catchment-level estimations of seroprevalences and PCR-based prevalences. As a result, the sample sizes of survey households per catchment were highly varied between health facilities, with one facility's records being excluded from this study as zero survey households were located within the estimated catchment area. Secondly, seroprevalences and PCR-prevalences were calculated using different denominators to the RDT and microscopy case metrics, therefore are not directly comparable. The active surveillance survey metrics were calculated using samples per area, and the passive surveillance metrics were calculated per capita from the estimated population size per catchment (3). Some households may also utilize near-by health facilities that are not the administratively assigned ones for their households, resulting in discrepancies in the catchment-area population estimations. While it is useful to visualize these various diagnostic endpoints on the same map (Figure 4), as they can help to pick up broad patterns in recent and current exposure, these limitations should be considered when interpreting these results.

Despite these limitations, we have shown that adding serology into passive surveillance projects can provide additional information on current and historic trends in exposure. The higher numbers of *P. vivax* compared to *P. falciparum* cases detected in the passive and active surveillance were reflected in the seroprevalence estimates for northern Lao PDR from this study, indicating that at a broad level, our serological estimations reflect the epidemiology of malaria in the area. We also demonstrated the use of health facility data to contextualize findings from serological burden estimations. The addition of serology in this study allowed for the characterization of the spatial distributions of exposures to *P. vivax* and *P. falciparum*, demonstrating how these methods can provide valuable information for control and elimination programmes which need to identify and target remaining foci of infection in low transmission settings. Additionally, we showed how active surveillance data can be linked to passive surveillance data, and the challenges which come with this. Future work should prioritize spatial and temporal alignment of sampling wherever possible, with design and implementation of user-friendly platforms to move these analyses into more routine public health use.

## Data availability statement

The datasets used and/or analyzed during the current study are available from the corresponding author on reasonable request.

## Ethics statement

The studies involving human participants were reviewed and approved by University of California San Francisco. Written informed consent to participate in this study was provided by the participants' legal guardian/next of kin.

## Author contributions

IB: investigation, formal analysis, and writing—original draft. EC, LN, and ED: support for analysis. EC, LN, FR, and ED: data interpretation. LN, FR, and BH: writing—review. LN, GS, AB, CD, and LW: conceptualization. FR and LW: data curation. CP and JR: conducting serological analyses. CP: completing quality control and normalization. KT: supporting laboratory analysis of serological samples. ED, GS, AL, AB, and CD: writing—review and editing. KF, GS, AL, and CD: supervision. All authors contributed to the article and approved the submitted version.

## Funding

Funding for the collection of data used in study was provided to the MEI at the UCSF by the Bill and Melinda Gates Foundation (OPP# 1116450). This study was funded by the Bill and Melinda Gates Foundation (OPP# 1177272).

## Conflict of interest

The authors declare that the research was conducted in the absence of any commercial or financial relationships that could be construed as a potential conflict of interest.

## Publisher's note

All claims expressed in this article are solely those of the authors and do not necessarily represent those of their affiliated organizations, or those of the publisher, the editors and the reviewers. Any product that may be evaluated in this article, or claim that may be made by its manufacturer, is not guaranteed or endorsed by the publisher.
