# Supplementary material for: Characterizing the spatial distribution of multiple malaria diagnostic endpoints in a low-transmission setting in Lao PDR
Source: Front Med (Lausanne). 2022 Aug 18;9:929366. doi: 10.3389/fmed.2022.929366 (PMC9433740; doi:10.3389/fmed.2022.929366)
Supplement: Supplementary file 1 [file Data_Sheet_1.PDF]

Supplementary Info

Supplementary Figure 1a & 1b: Scatterplots showing MFI values for each antigen used to define seropositivity, by age. Figure 1a shows P. vivax antigens and 1b shows P. falciparum antigens. This represents the full active survey population (n = 5082).

Supplementary Figure 2: Maps of upper and lower limits of 95% credible interval of exposures. Maps created using QGIS.

Supplementary Figure 3: Left: Health centre locations used to derive catchment areas. Right: Catchment areas derived using health centre locations and travel time friction surface. The catchments filled red are the health centres which the passive surveillance data were collected from. Maps created using QGIS

**Species**	**Antigen**	**Description**	**Plasmodb ID**	**Reference**
P.falciparum	PfAMA1	Apical membrane antigen 1	PF3D7_1133400	(1)
P.falciparum	PfMSP1_19	Merozoite surface protein 1-19	PF3D7_0930300	(2)
P.falciparum	GLURP R2	Glutamate-rich protein	PF3D7_1035300	(3)
P.falciparum	MSP2 CH150/9	Merozoite surface protein 2 full-length [CH150/9]	PF3D7_0206800	(4)
P.falciparum	MSP2 Dd2	Merozoite surface protein 2 full-length [Dd2]	PF3D7_0206800	(4)
P.falciparum	Etramp 4 Ag 2	Early transcribed membrane protein 4 antigen (exon) 2	PF3D7_0423700	(5); Tetteh K unpublished
P.falciparum	Etramp 5 Ag 1	Early transcribed membrane protein 5 antigen (exon) 1	PF3D7_0532100	(5); Tetteh K unpublished
P.falciparum	GEXP18	Plasmodium exported protein	PF3D7_0402400	(6)
P.falciparum	HSP40 Ag 1	Heat shock protein 40, type II	PF3D7_0501100.1	(6)
P.falciparum	Hyp 2	Plasmodium exported protein (hyp2)	PF3D7_1002000	(6)
P.falciparum	PfSEA-1	Schizont egress antigen-1	PF3D7_1021800	(7); Tetteh K unpublished
P.falciparum	SBP1	Skeleton-binding protein 1	PF3D7_0501300	(8); Tetteh K unpublished
P.falciparum	Rh2_2030	Reticulocyte binding protein 2 homologue a	PF3D7_1335400	(9)
P.falciparum	Rh4.2	Reticulocyte binding protein homologue 4 (RH4)	PF3D7_0424200	(10)
P.falciparum	Rh5.1	Reticulocyte binding protein homologue 5	PF3D7_0424100	(11);Tetteh K unpublished
P.falciparum	EBA140 RIII-V	Erythrocyte binding antigen-140	PF3D7_1301600	(12)
P.falciparum	EBA175 RIII-V	Erythrocyte binding antigen-175	PF3D7_0731500	(12)
P.falciparum	EBA181 RIII-V	Erythrocyte binding antigen-181	PF3D7_0102500	(12)
P.falciparum	CSP	Circumsporozoite protein	PF3D7_0304600	(13)
P.vivax	PvMSP119	Merozoite surface protein 1-19	PVX_099980	(14,15)
P.vivax	PvAMA1	Apical membrane antigen 1	PVX_092275	(16)
P.vivax	PvDBPRII	Duffy-binding protein RII	PVX_110810	(17,18)
P.vivax	PvEBPII	*P. vivax* erythrocyte binding protein	PVX_110810	(19–21)
P.vivax	PvRBP1a	*P. vivax* reticulocyte binding protein *1a*	PVX_098585	(22,23)
P.vivax	PvRBP2b	*P. vivax* reticulocyte binding protein *1b*	PVX_094255	(22,23)

Supplementary Information Table 1: Full list of malaria antigens run on Luminex MBA, broken down by Plasmodium species

Supplementary Information Table 2: Environmental and spatial covariates

Parameter	Description	Resolution	Time period	Source
Elevation	Elevation (metres above sea level)	30 m	2019	ASTER Global Digital Elevation Map (24)
Slope and aspect	Slope and aspect (degrees)	30 m	2016	Calculated from ASTER Global Digital Elevation Map (24)
TWI	Topographic wetness index	30m	2016	Calculated from ASTER Global Digital Elevation Map (24)
EVI & NDVI	Enhanced Vegetation Index & Normalised differential vegetation index	30 m	Sept – Oct 2016	NASA Terra MODIS (25)
Land cover	Euclidean distance to different land cover types	10 m	2017	Calculated from land cover map(26)
Location of houses	GPS coordinates	5 m		Mapped during GPS field surveys(27)
Distance to roads and houses	Distance from nearest road	10 m	2016	Calculated from shapefile of roads from(28)
Accessibility to nearest hospital	Global map of accessibilities to health facilities	1000m	2017	Global map of accessibilities to health

				facilities(29)
Population density	UN-adjusted 2015 population density	100 m	2015	World Pop (30)
Bioclimatic variables	Bioclimatic indicators of ecology	1000 m	1970 - 2000	Calculated from (31)

Supplementary Information Table 3:

a. Historic Pv Exposure

Covariate*	Mean	95% Bayesian Credible Interval (BCI)	
		2.5%	97.5%
Annual temperature range	0.299	0.098	0.535
Distance from built environment	0.079	-0.071	0.233
Distance from roads	0.157	0.038	0.275
Slope	-0.123	-0.242	-0.005

* All covariates mean-centered and squared

b. Recent Pv Exposure

Covariate*	Mean	95% Bayesian Credible Interval (BCI)	
		2.5%	97.5%
Elevation	-0.391	-0.698	-0.1
Distance from roads	-0.047	-0.281	-0.041
Treecover	-0.144	-0.359	-0.143

* All covariates mean-centered and squared

c. Historic Pf Exposure

Covariate*	Mean	95% Bayesian Credible Interval (BCI)	
		2.5%	97.5%
Annual temperature range	0.499	-0.130	1.107
Distance from built environment	-0.169	-0.523	-0.157

* All covariates mean-centered and squared

d. Recent Pv Exposure

Covariate*	Mean	95% Bayesian Credible Interval (BCI)	
		2.5%	97.5%
Mean diurnal temperature range	0.543	-1.282	2.556
Distance from bare soil	-0.786	-1.658	-0.076

* All covariates mean-centered and squared

Supplementary information references

1. Collins CR, Withers-Martinez C, Bentley GA, Batchelor AH, Thomas AW, Blackman MJ. Fine mapping of an epitope recognized by an invasion-inhibitory monoclonal antibody on the malaria vaccine candidate apical membrane antigen 1. J Biol Chem [Internet]. 2007 Mar 2 [cited 2022 Jun 9];282(10):7431–41. Available from: https://pubmed.ncbi.nlm.nih.gov/17192270/

2. Burghaus PA, Holder AA. Expression of the 19-kilodalton carboxy-terminal fragment of the Plasmodium falciparum merozoite surface protein-1 in Escherichia coli as a correctly folded protein. Mol Biochem Parasitol [Internet]. 1994 [cited 2022 Jun 9];64(1):165–9. Available from: https://pubmed.ncbi.nlm.nih.gov/8078519/

3. Theisen M, Vuust J, Gottschau A, Jepsen S, Hogh B. Antigenicity and immunogenicity of recombinant glutamate-rich protein of Plasmodium falciparum expressed in Escherichia coli. Clin Diagn Lab Immunol [Internet]. 1995 [cited 2022 Jun 9];2(1):30–4. Available from: https://pubmed.ncbi.nlm.nih.gov/7719909/

4. Polley SD, Conway DJ, Cavanagh DR, McBride JS, Lowe BS, Williams TN, et al. High levels of serum antibodies to merozoite surface protein 2 of Plasmodium falciparum are associated with reduced risk of clinical malaria in coastal Kenya. Vaccine [Internet]. 2006 May 8 [cited 2022 Jun 9];24(19):4233–46. Available from: https://pubmed.ncbi.nlm.nih.gov/16111789/

5. Spielmann T, Fergusen DJP, Beck HP. etramps, a new Plasmodium falciparum gene family coding for developmentally regulated and highly charged membrane proteins located at the parasite-host cell interface. Mol Biol Cell [Internet]. 2003 Apr 1 [cited 2022 Jun 9];14(4):1529–44. Available from: https://pubmed.ncbi.nlm.nih.gov/12686607/

6. Helb DA, Tetteh KKA, Felgner PL, Skinner J, Hubbard A, Arinaitwe E, et al. Novel serologic biomarkers provide accurate estimates of recent Plasmodium falciparum exposure for individuals and communities. Proc Natl Acad Sci U S A [Internet]. 2015 Aug 11 [cited 2022 Mar 29];112(32):E4438–47. Available from: /pmc/articles/PMC4538641/

7. Raj DK, Nixon CP, Nixon CE, Dvorin JD, DiPetrillo CG, Pond-Tor S, et al. Antibodies to PfSEA-1 block parasite egress from RBCs and protect against malaria infection. Science [Internet]. 2014 [cited 2022 Jun 9];344(6186):871–7. Available from: https://pubmed.ncbi.nlm.nih.gov/24855263/

8. Grüring C, Heiber A, Kruse F, Ungefehr J, Gilberger TW, Spielmann T. Development and host cell modifications of Plasmodium falciparum blood stages in four dimensions. Nat Commun [Internet]. 2011 [cited 2022 Jun 9];2(1). Available from: https://pubmed.ncbi.nlm.nih.gov/21266965/

9. Triglia T, Thompson J, Caruana SR, Delorenzi M, Speed T, Cowman AF. Identification of proteins from Plasmodium falciparum that are homologous to reticulocyte binding proteins in Plasmodium vivax. Infect Immun [Internet]. 2001 [cited 2022 Jun 9];69(2):1084–92. Available from: https://pubmed.ncbi.nlm.nih.gov/11160005/

10. Reiling L, Richards JS, Fowkes FJI, Wilson DW, Chokejindachai W, Barry AE, et al. The Plasmodium falciparum erythrocyte invasion ligand Pfrh4 as a target of functional and protective human antibodies against malaria. PLoS One [Internet]. 2012 Sep 20 [cited 2022 Jun 9];7(9). Available from: https://pubmed.ncbi.nlm.nih.gov/23028883/

11. Crosnier C, Bustamante LY, Bartholdson SJ, Bei AK, Theron M, Uchikawa M, et al. Basigin is a receptor essential for erythrocyte invasion by Plasmodium falciparum. Nature [Internet]. 2011 Dec 22 [cited 2022 Jun 9];480(7378):534–7. Available from:

https://pubmed.ncbi.nlm.nih.gov/22080952/

12. Richards JS, Stanisic DI, Fowkes FJI, Tavul L, Dabod E, Thompson JK, et al. Association between naturally acquired antibodies to erythrocyte-binding antigens of Plasmodium falciparum and protection from malaria and high-density parasitemia. Clin Infect Dis [Internet]. 2010 Oct 15 [cited 2022 Jun 9];51(8). Available from: https://pubmed.ncbi.nlm.nih.gov/20843207/

13. Kastenmüller K, Espinosa DA, Trager L, Stoyanov C, Salazar AM, Pokalwar S, et al. Full-length Plasmodium falciparum circumsporozoite protein administered with long-chain poly(I·C) or the Toll-like receptor 4 agonist glucopyranosyl lipid adjuvant-stable emulsion elicits potent antibody and CD4+ T cell immunity and protection in mice. Infect Immun [Internet]. 2013 Mar [cited 2022 Jun 9];81(3):789–800. Available from: https://pubmed.ncbi.nlm.nih.gov/23275094/

14. Cunha MG, Rodrigues MM, Soares IS. Comparison of the immunogenic properties of recombinant proteins representing the Plasmodium vivax vaccine candidate MSP1(19) expressed in distinct bacterial vectors. Vaccine [Internet]. 2001 Nov 12 [cited 2022 Jun 15];20(3–4):385–96. Available from: https://pubmed.ncbi.nlm.nih.gov/11672901/

15. Soares IS, Barnwell JW, Ferreira MU, Da Cunha MG, Laurino JP, Castilho BA, et al. A Plasmodium vivax vaccine candidate displays limited allele polymorphism, which does not restrict recognition by antibodies. Mol Med [Internet]. 1999 [cited 2022 Jun 15];5(7):459. Available from: /pmc/articles/PMC2230447/?report=abstract

16. Kocken CHM, Dubbeld MA, Van Der Wel A, Pronk JT, Waters AP, Langermans JAM, et al. High-level expression of Plasmodium vivax apical membrane antigen 1 (AMA-1) in Pichia pastoris: strong immunogenicity in Macaca mulatta immunized with P. vivax AMA-1 and adjuvant SBAS2. Infect Immun [Internet]. 1999 [cited 2022 Jun 14];67(1):43–9. Available from: https://pubmed.ncbi.nlm.nih.gov/9864194/

17. King CL, Michon P, Shakri AR, Marcotty A, Stanisic D, Zimmerman PA, et al. Naturally acquired Duffy-binding protein-specific binding inhibitory antibodies confer protection from blood-stage Plasmodium vivax infection. Proc Natl Acad Sci U S A [Internet]. 2008 Jun 6 [cited 2022 Jun 14];105(24):8363. Available from: /pmc/articles/PMC2448842/

18. Grimberg BT, Udomsangpetch R, Xainli J, McHenry A, Panichakul T, Sattabongkot J, et al. Plasmodium vivax Invasion of Human Erythrocytes Inhibited by Antibodies Directed against the Duffy Binding Protein. PLoS Med [Internet]. 2007 Dec [cited 2022 Jun 14];4(12):1940–8. Available from: /pmc/articles/PMC2140086/

19. Hester J, Chan ER, Menard D, Mercereau-Puijalon O, Barnwell J, Zimmerman PA, et al. De Novo Assembly of a Field Isolate Genome Reveals Novel Plasmodium vivax Erythrocyte Invasion Genes. PLoS Negl Trop Dis [Internet]. 2013 [cited 2022 Jun 14];7(12):e2569. Available from: https://journals.plos.org/plosntds/article?id=10.1371/journal.pntd.0002569

20. Menard D, Chan ER, Benedet C, Ratsimbasoa A, Kim S, Chim P, et al. Whole Genome Sequencing of Field Isolates Reveals a Common Duplication of the Duffy Binding Protein Gene in Malagasy Plasmodium vivax Strains. PLoS Negl Trop Dis [Internet]. 2013 [cited 2022 Jun 14];7(11):e2489. Available from: https://journals.plos.org/plosntds/article?id=10.1371/journal.pntd.0002489

21. Roesch C, Popovici J, Bin S, Run V, Kim S, Ramboarina S, et al. Genetic diversity in two Plasmodium vivax protein ligands for reticulocyte invasion. PLoS Negl Trop Dis [Internet]. 2018 Oct 1 [cited 2022 Jun 14];12(10):e0006555. Available from: https://journals.plos.org/plosntds/article?id=10.1371/journal.pntd.0006555

22. Hietanen J, Chim-Ong A, Chiramanewong T, Gruszczyk J, Roobsoong W, Tham WH, et al. Gene Models, Expression Repertoire, and Immune Response of Plasmodium vivax Reticulocyte Binding Proteins. Infect Immun [Internet]. 2016 Mar 1 [cited 2022 Jun 14];84(3):677. Available from: /pmc/articles/PMC4771344/

23. França CT, He WQ, Gruszczyk J, Lim NTY, Lin E, Kiniboro B, et al. Plasmodium vivax Reticulocyte Binding Proteins Are Key Targets of Naturally Acquired Immunity in Young Papua New Guinean Children. PLoS Negl Trop Dis [Internet]. 2016 Sep 27 [cited 2022 Jun 14];10(9):e0005014. Available from: https://journals.plos.org/plosntds/article?id=10.1371/journal.pntd.0005014

24. NASA/METI/AIST/Japan Spacesystems and US /Japa. AST. ASTER Global Digital Elevation Model V003 [Data set]. [Internet]. NASA EOSDIS Land Processes DAAC. 2019 [cited 2020 Jul 14]. Available from: https://lpdaac.usgs.gov/products/astgtmv003

25. Didan K. MOD13Q1 MODIS/Terra Vegetation Indices 16-Day L3 Global 250m SIN Grid V006. NASA EOSDIS Land Processes DAAC. [Internet]. Vol. 5, NASA EOSDIS Land Processes DAAC. 2015 [cited 2020 Jun 4]. p. 2002–15. Available from: http://doi.org/10.5067/MODIS/MOD13Q1.006

26. Kontgis K. Global land use/land cover with Sentinel-2 and deep learning. In: IGARSS 2021-2021 IEEE International Geoscience and Remote Sensing Symposium [Internet]. IEEE; 2021 [cited 2022 Mar 25]. Available from: https://www.arcgis.com/home/item.html?id=d6642f8a4f6d4685a24ae2dc0c73d4ac

27. Lover AA, Dantzer E, Hongvanthong B, Chindavongsa K, Welty S, Reza T, et al. Prevalence and risk factors for asymptomatic malaria and genotyping of glucose 6-phosphate (G6PD) deficiencies in a vivax-predominant setting, Lao PDR: implications for sub-national elimination goals. Malar J [Internet]. 2018 Jun 1 [cited 2022 Mar 24];17(1). Available from: https://pubmed.ncbi.nlm.nih.gov/29859089/

28. Road Networks in Laos 2021 (OpenStreetMap) - Dataset OD Mekong Datahub [Internet]. [cited 2022 Mar 25]. Available from: https://data.opendevelopmentmekong.net/dataset/road-networks-in-laos-2021-openstreetmap

29. Weiss DJ, Nelson A, Vargas-Ruiz CA, Gligorić K, Bavadekar S, Gabrilovich E, et al. Global maps of travel time to healthcare facilities. Nat Med [Internet]. 2020;26(December). Available from: http://dx.doi.org/10.1038/s41591-020-1059-1

30. Lloyd CT, Sorichetta A, Tatem AJ. High resolution global gridded data for use in population studies. Sci Data 2017 41 [Internet]. 2017 Jan 31 [cited 2022 Mar 25];4(1):1–17. Available from: https://www.nature.com/articles/sdata20171

31. Fick SE, Hijmans RJ. WorldClim 2: new 1-km spatial resolution climate surfaces for global land areas. Int J Climatol [Internet]. 2017 Oct 1 [cited 2022 Mar 25];37(12):4302–15. Available from: https://onlinelibrary.wiley.com/doi/full/10.1002/joc.5086
