# Supplementary figures and images for: Characterizing the spatial distribution of multiple malaria diagnostic endpoints in a low-transmission setting in Lao PDR
Source: Front Med (Lausanne). 2022 Aug 18;9:929366. doi: 10.3389/fmed.2022.929366 (PMC9433740; doi:10.3389/fmed.2022.929366)

A)

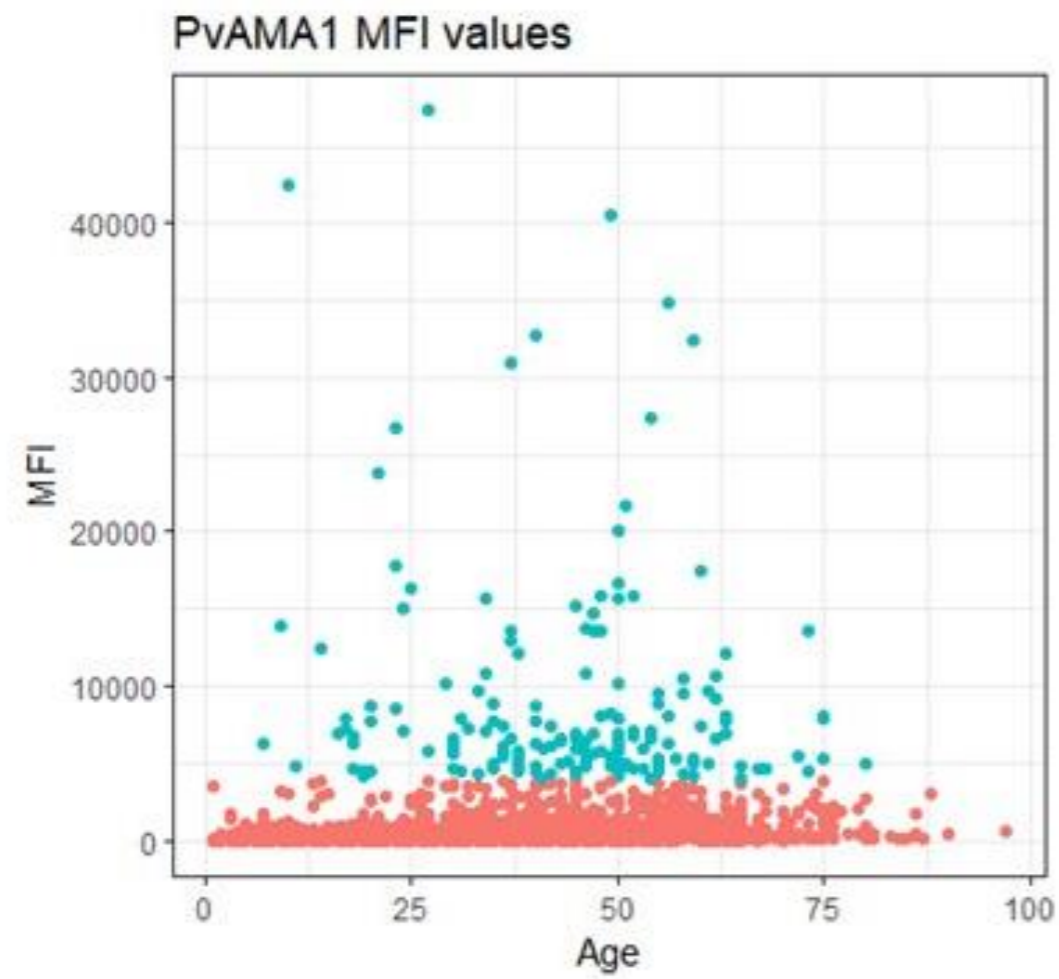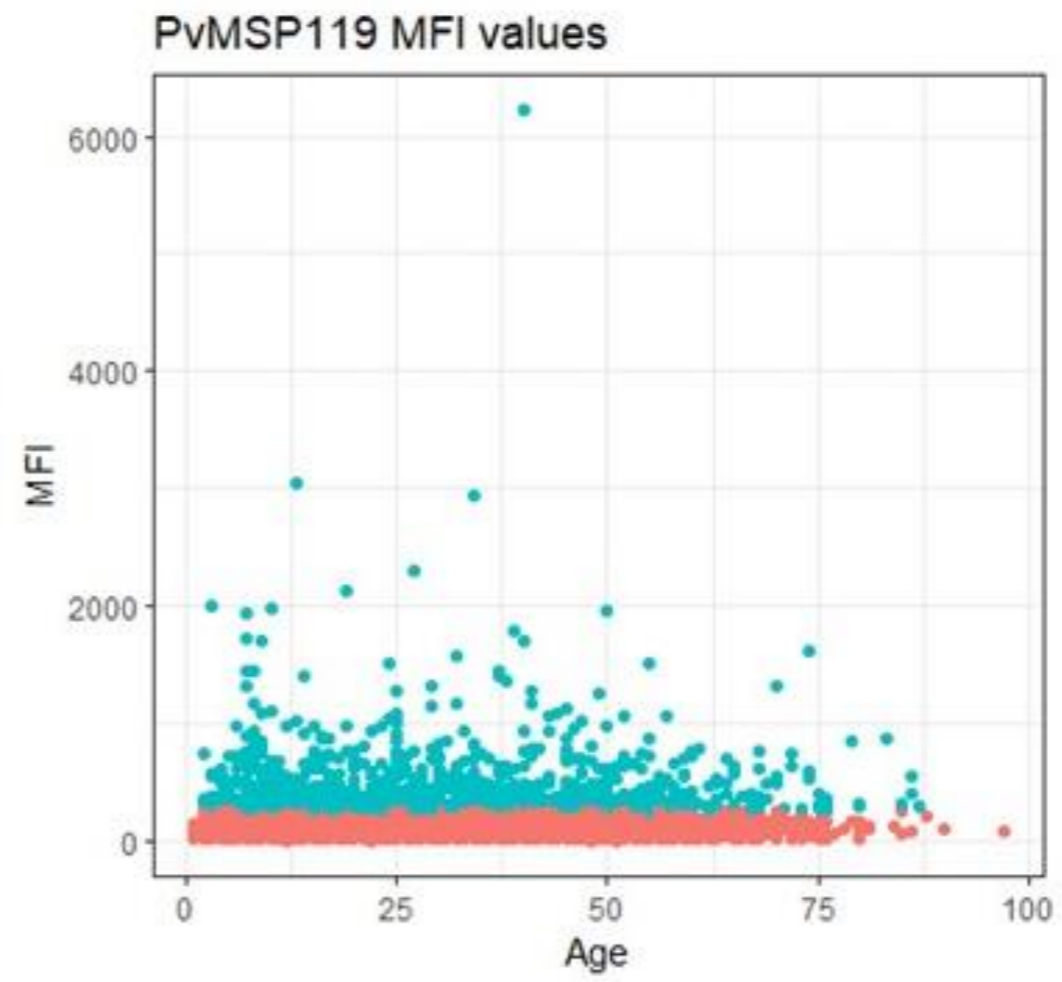

B)

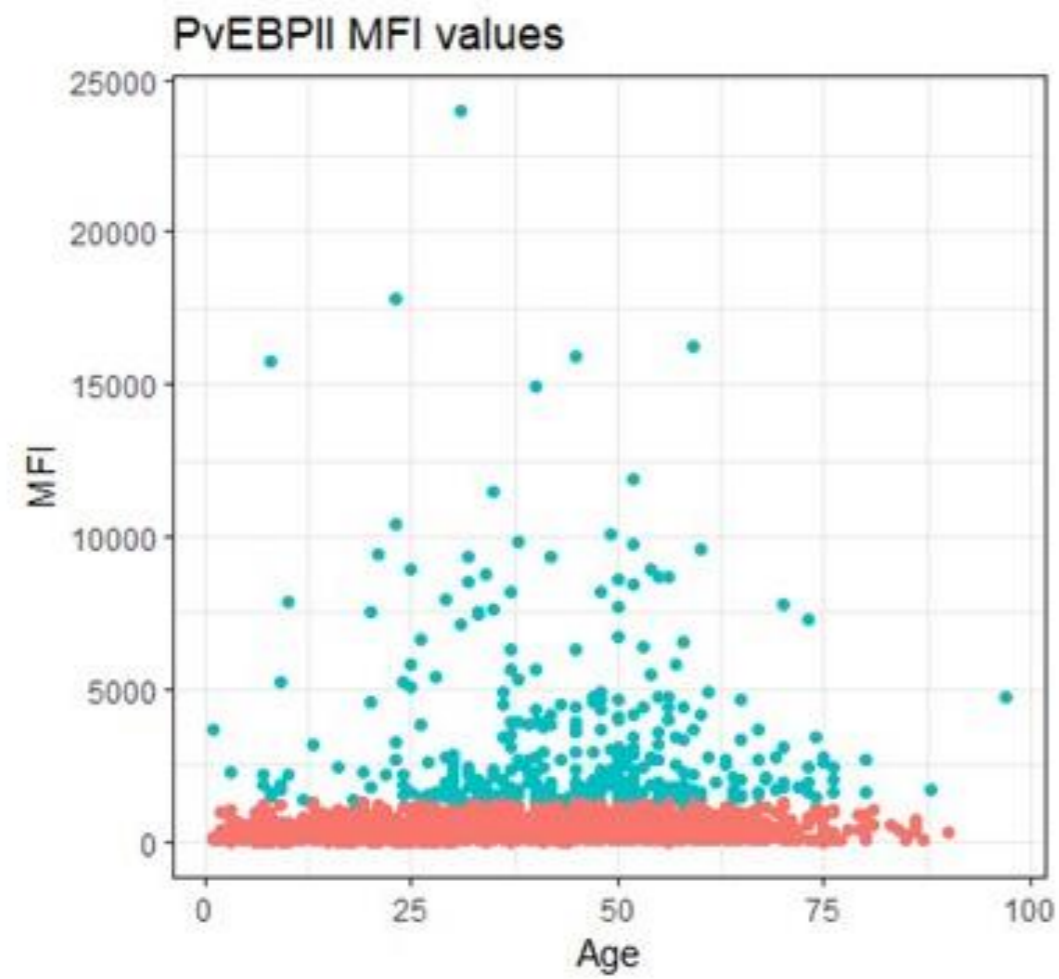

B)

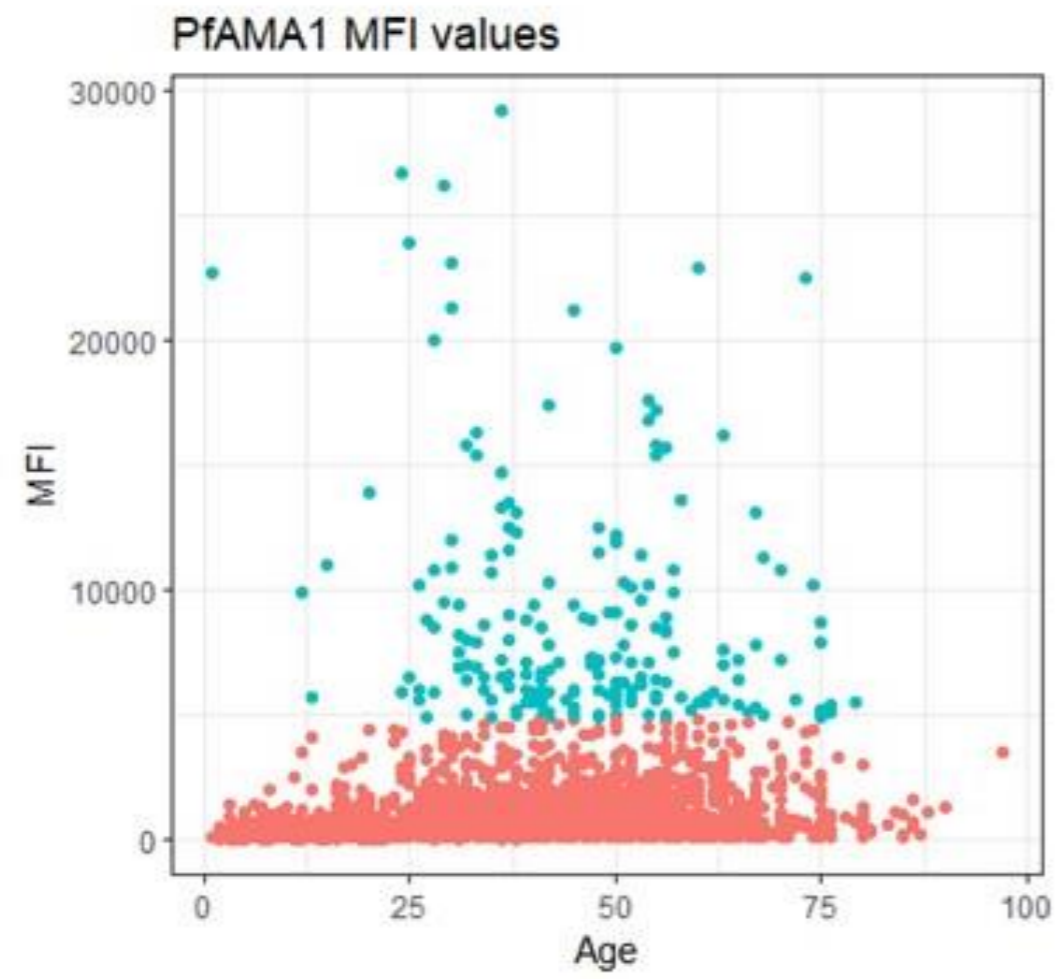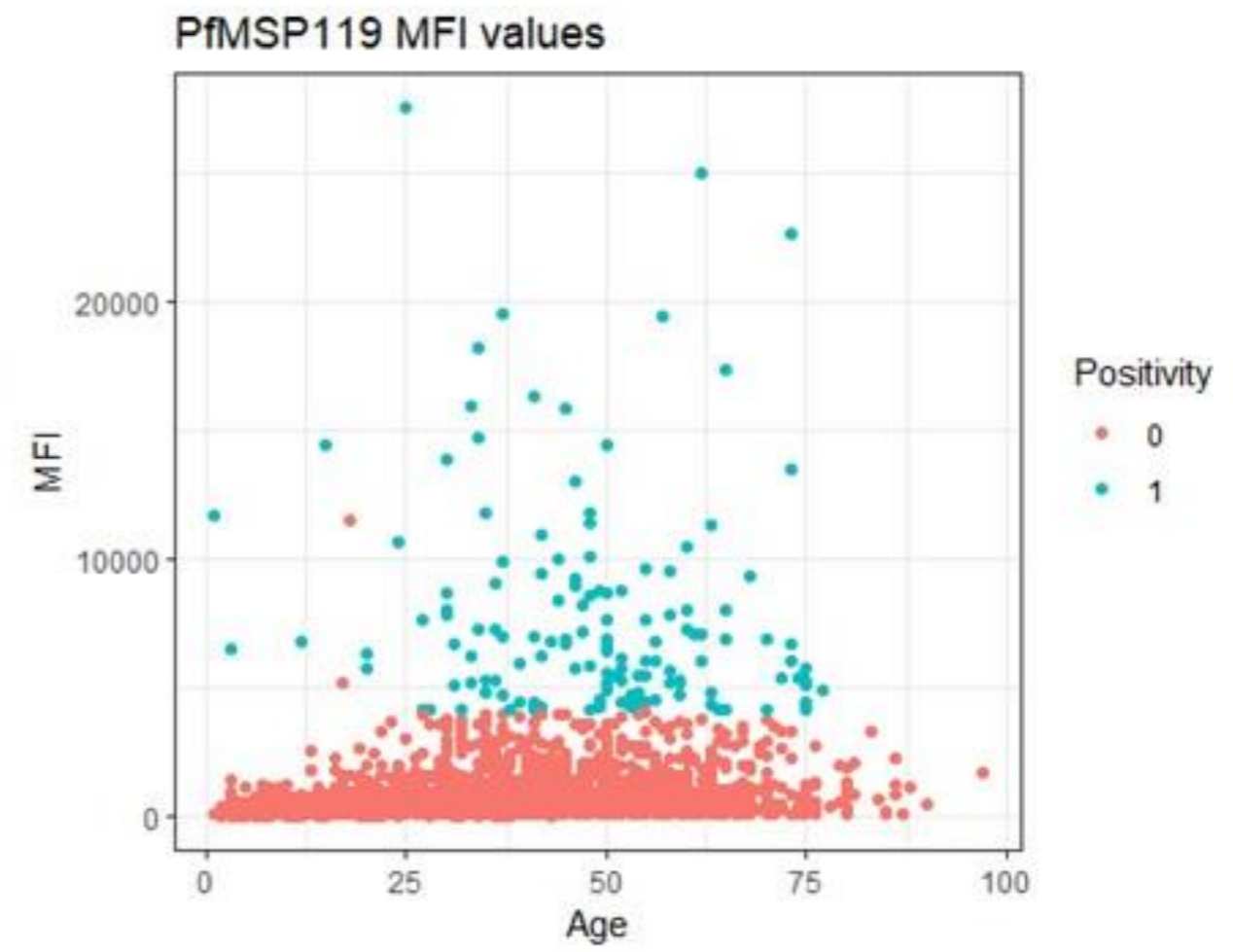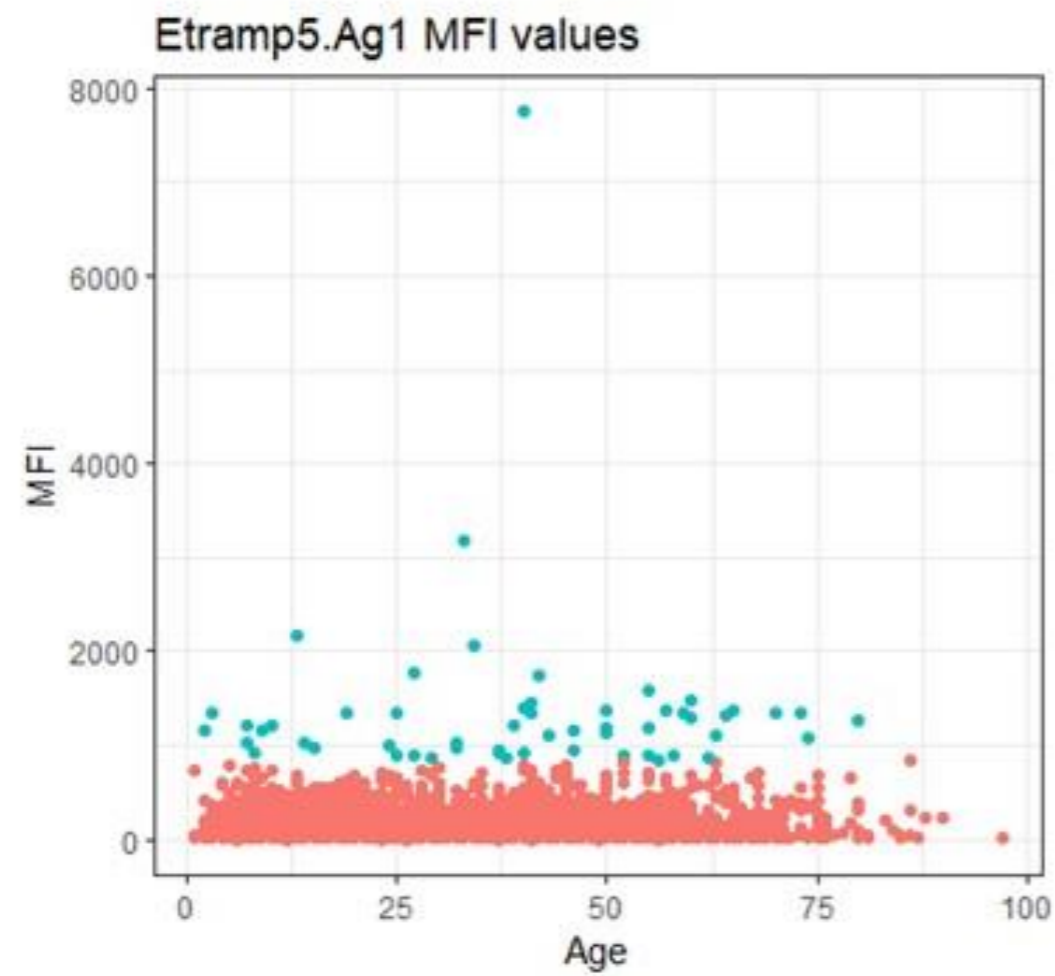

Supplement: Supplementary file 2 [file Image_1.pdf]

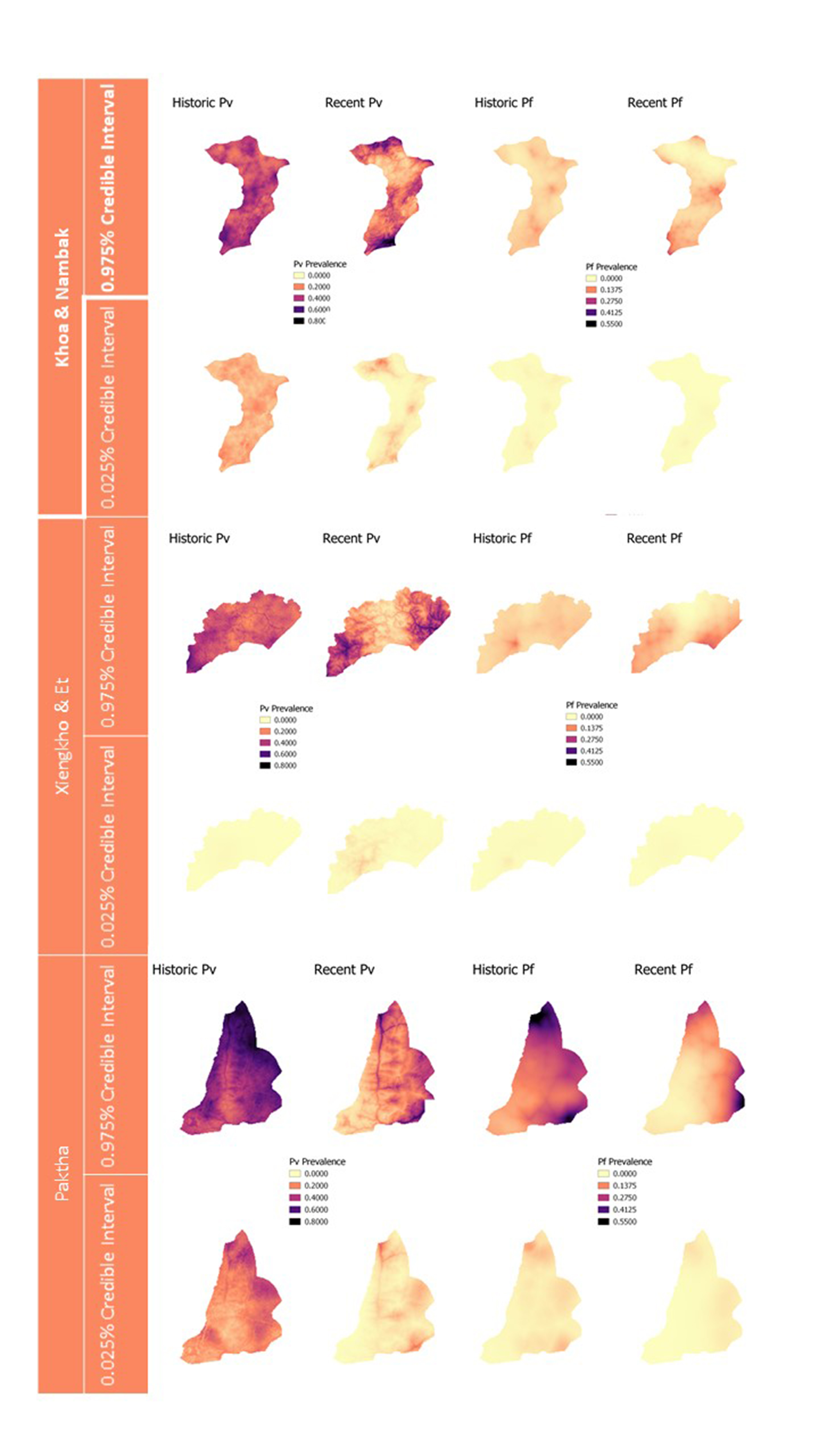

Supplement: Supplementary file 3 [file Image_2.jpeg]

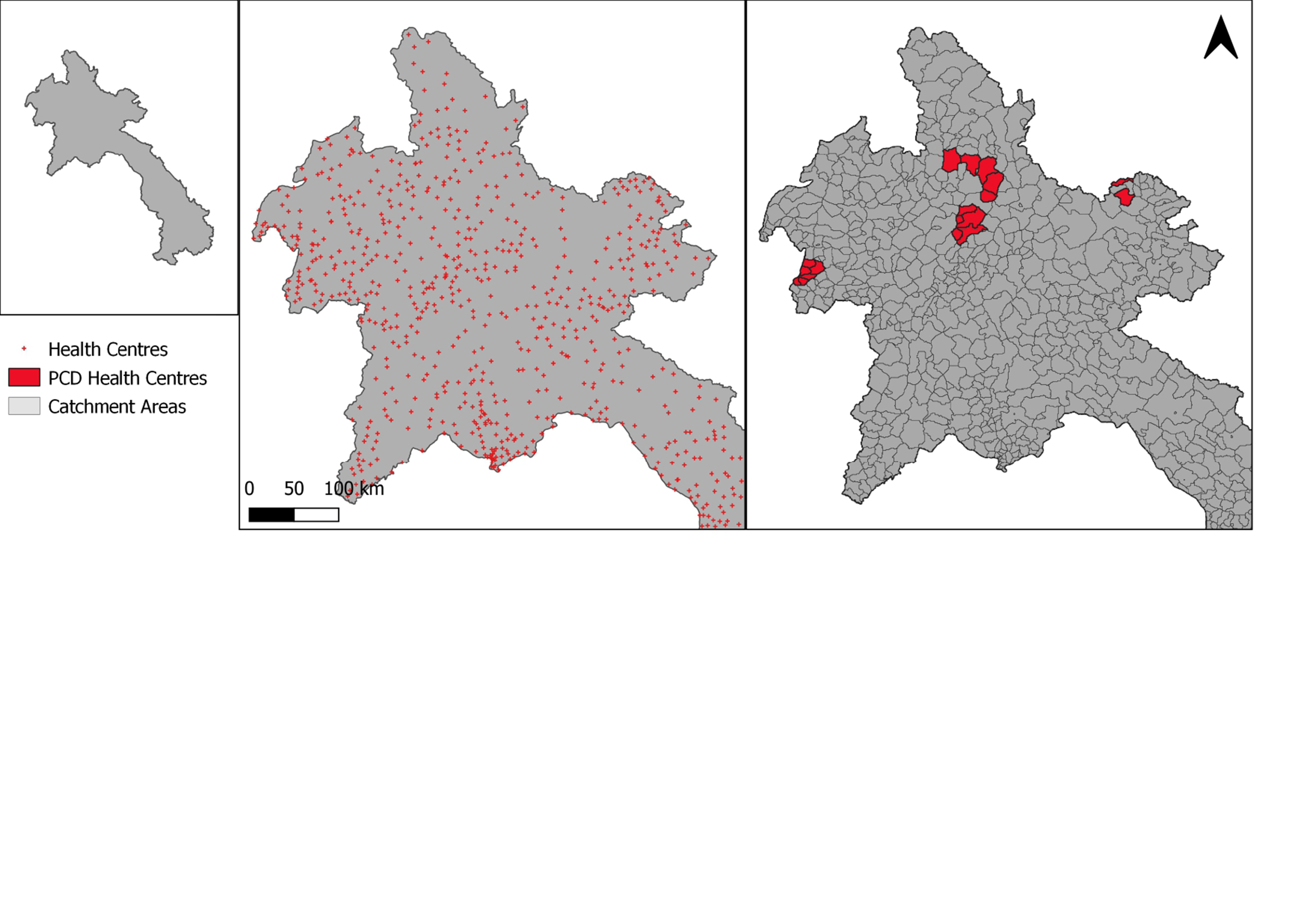

Supplement: Supplementary file 4 [file Image_3.jpeg]
